# A Case of Progestogen Hypersensitivity: Systemic Allergic Response

**DOI:** 10.7759/cureus.92007

**Published:** 2025-09-10

**Authors:** Hannah R Smith, Kyler Stephan, Larry M Fields

**Affiliations:** 1 Obstetrics and Gynecology, Lincoln Memorial University DeBusk College of Osteopathic Medicine, Knoxville, USA; 2 Obstetrics and Gynecology, Tennova Turkey Creek Medical Center, Knoxville, USA

**Keywords:** allergic hypersensitivity, progesterone, progestin, progestogen, systemic allergic conditions

## Abstract

Progestogen hypersensitivity (PH) is an allergic condition in which women of reproductive age become sensitized to endogenous progesterone and/or exogenous progestins. PH may present as cutaneous manifestations, such as hives, and/or systemic allergic reactions. Allergic flares typically correlate with relative peaks in progesterone during the luteal phase of the menstrual cycle.PH may go unrecognized due to its heterogeneous presentation. In this case report, we discuss a 26-year-old female patient with suspected progestogen hypersensitivity resulting in a monthly systemic allergic response. We also discuss diagnostic factors for PH, pathogenesis, and treatment/management of symptoms.

## Introduction

Progestogens are endogenous or synthetic steroid sex hormones that play a role in menstrual cycles and the maintenance of pregnancy. Progesterone is initially produced by the ovaries in low levels, but after the luteinizing hormone (LH) surge and ovulation, typically on day 14 in a standard menstrual cycle, production is taken over by the corpus luteum. An oocyte is released from a ruptured follicle, which becomes the corpus luteum and is responsible for the majority of progesterone production during the luteal phase, with peak levels occurring in the mid to late luteal phase. In pregnancy, the corpus luteum is responsible for the initial progesterone that helps to maintain the pregnancy until the placenta has developed enough. The placenta produces progesterone and will eventually take over the main role of producing progesterone to maintain a pregnancy [1].

Allergic reactions, also known as hypersensitivity reactions, result from exposure to a novel foreign substance. In Type I hypersensitivity reactions, antigens are taken up by antigen-presenting cells (APCs) and presented to T helper 2 cells, and after initial exposure, antigen-specific IgE antibodies are formed. With subsequent antigen exposure, antigens bind their specific IgE antibodies, and this complex binds to and activates mast cells and basophils with a subsequent release of histamines, prostaglandins, and leukotrienes, all with vasodilating effects to achieve the end goal of an inflammatory response. This presents clinically with anaphylaxis, hypotension, angioedema, bronchospasm, and urticaria. In Type 4, or cell-mediated, hypersensitivity reactions, antigen exposure and presentation activate T helper 1 cell differentiation. Upon re-exposure, the Th1 cells release cytokines that recruit and activate cytotoxic T cells and macrophages. This most commonly presents clinically with contact dermatitis. Drug-induced hypersensitivity syndrome (DiHS) is a severe subtype that presents with rash, fever, and multiorgan involvement [2].

Progestogen hypersensitivity (PH) is associated with exposure to exogenous progestins or endogenous progesterone and has been theorized to develop as a Type I or Type IV hypersensitivity reaction due to its positive skin test and intramuscular challenges to progestogens [3]. People with PH can present with a wide array of cutaneous manifestations, including urticaria, angioedema, erythema multiforme, eczema, folliculitis, papulovesicular eruptions, purpura, or vulvovaginal pruritus. PH is most commonly seen with prior exogenous progestin exposure, usually in the form of oral contraceptive pills, but in some cases it manifests in correlation with menstrual cycles, during the luteal phase, with peaks of endogenous progesterone production [4]. This report describes the case of a 26-year-old female patient with progressively worsening pruritus, rash, swelling, and GI upset associated with menstrual cycles over a six-month period.

## Case presentation

A 26-year-old female patient (G2P2002) presented to an outpatient gynecology clinic for evaluation of cyclic reactive episodes corresponding to menses. Her gynecologic history is remarkable for previous concerns for an increase in acne as well as symptoms of dysmenorrhea and oligomenorrhea. A pelvic ultrasound was performed eight months prior to presentation and suggested polycystic ovarian changes, per physician documentation. She reported two previous uncomplicated spontaneous vaginal deliveries. She was currently not taking any hormonal medications and had no previous history of hormonal contraceptive use.

The patient reported intermittent two to five-day episodes of allergic-type symptoms (diffuse itching, rash, facial swelling, increased heart rate) in addition to progressively worsening gastrointestinal upset (nausea, vomiting, diarrhea, and increased pelvic cramping) during menses or just after menses for the last six months. Benadryl helped initially, but it is no longer effective. She presented to the emergency room multiple times during these episodes for difficulty breathing and wheezing. She was stabilized during these ED visits and sent home with a prescription for an EpiPen, which provided minimal relief. She tried a progesterone-based oral contraceptive pill for several months, and this increased the number and intensity of episodes. 

The patient had previously seen her primary care physician, an allergist, and obtained a CT abdomen/pelvis for evaluation prior to presentation to an outpatient gynecology clinic; ultimately ruling out mast cell syndrome, serotonin syndrome, and any external exposure to an offending trigger. It is unclear if the allergist did progesterone-specific testing. We were unable to ascertain from the documentation from the allergist or from the patient herself. The CT abdomen/pelvis results were confirmed with the patient’s primary care physician and allergist via telephone communication. Based on previous workup, the patient was advised to follow up with gynecology, avoid progesterone-based contraceptives, and monitor symptoms. 

At her presentation at the outpatient gynecology clinic, several labs were ordered, and the results are listed in Table 1. 

**Table 1:** Laboratory test results DHEA: dehydroepiandrosterone

Parameters	Patient Value	Reference Range
DHEA-Sulfate (µg/dL)	309	99-340
Estradiol (pg/mL)	43	Healthy women: Follicular Phase: 12.4-233; Ovulation Phase: 41.0-398; Luteal Phase: 22.3-341; Postmenopause: <5-138; Healthy pregnant women: 1st Trimester: 154-3243; 2nd Trimester: 1561-21280; 3rd Trimester: 8525->30000
Progesterone (ng/mL)	0.23	Healthy women: Follicular Phase: 0.057-0.893; Ovulation Phase: 0.121-12.0; Luteal Phase: 1.83-23.9; Postmenopause: <0.05-0.126; Healthy Pregnant Women: 1st Trimester: 11.0-44.3; 2nd Trimester: 25.4-83.3; 3rd Trimester: 58.7-214
Follicle-Stimulating Hormone (mIU/mL)	6.60	Women: Follicular Phase: 3.5-12.5; Ovulation Phase: 4.7-21.5; Luteal Phase: 1.7-7.7; Postmenopause: 25.8-134.8
Luteinizing Hormone (mIU/mL)	6.50	Women: Follicular Phase: 2.4-12.6; Ovulation Phase: 14.0-95.6; Luteal Phase: 1.0-11.4; Postmenopause: 7.7-58.5
Total Testosterone (ng/dL)	35.20	Women: 8.00-48.00
Thyroid-Stimulating Hormone (mU/L)	1.55	0.43-5.25
Free T3 (pg/mL)	3.30	2.30-4.40
Free Thyroxine (ng/dL)	1.37	0.86-1.76
Vitamin B12 (pg/mL)	638	232-1245
Vitamin D, 25-Hydroxy (ng/mL)	11.8	30.0-100.0

The correlation of symptoms with her menstrual cycles, with increasing symptom severity, meant that PH, although rare, was high on our differential. The outpatient gynecology clinic was in a small community hospital with minimal experience in diagnosing/treating these types of allergic reactions. At her follow-up appointment, her results were discussed extensively, and she was referred to a tertiary care center in a major nearby city for progesterone sensitivity testing and treatment. Unfortunately, the patient was lost to follow-up and has not been able to be contacted since her last follow-up.

## Discussion

PH is a systemic allergic response to exogenous and/or endogenous sources of progestogens. Upon reviewing the existing literature, the most common precipitating factor in the development of PH is prior exogenous progestin exposure. However, there are two overarching theories for why people develop PH. In one theory, it is proposed that synthetic progestins have added or modified side chains that are sufficiently different from natural progesterone to stimulate immunologic mechanisms facilitating subsequent hypersensitivity reactions to exogenous exposure [1,4]. The other theory can be used to explain the onset of PH in patients without a prior history of exogenous progestin exposure. It states that there is a possible cross-sensitivity of 17-alpha-hydroxyprogesterone with hydrocortisone, which can lead to the development of PH [4].

Reports have shown predominantly Type I and Type IV hypersensitivity reactions [5]. With exogenous sources, allergic response occurs following exposure, usually to oral contraceptive pills with progestin. With endogenous sources, allergic responses appear cyclical and are often associated with peak progesterone levels during the menstrual cycle. A case report in 1989 demonstrated progesterone’s effect on eosinophils and histamine release from mast cells, contributing to the clinical presentation of PH [1]. With further development of our understanding of hypersensitivity reactions and PH, we can now understand this case in the context of a type I hypersensitivity reaction.

In a type I hypersensitivity reaction, we see IgE antibody formation [5]. The IgE antibodies then bind mast cells, triggering histamine release, producing the symptoms of urticaria, angioedema, and anaphylaxis with subsequent prostaglandin, leukotriene, and eosinophil recruitment in the late phase of the reaction [4,6]. Type I hypersensitivity reactions usually happen after exposure to exogenous progesterone, typically with large doses such as those used in fertility treatments. Patients with positive skin testing to progesterone and basophil and mast cell activation likely have a type I hypersensitivity reaction [5]. Type IV hypersensitivity reactions in PH are supported by the delayed reactions to skin prick or intracutaneous testing with progesterone that have been seen in previous cases [5]. There are some inconsistent findings; while some cases have been related to hydrocortisone cross-sensitivity, others have been traced back to progesterone [7]. There have also been a few cases of type III hypersensitivity reactions in PH [8]. This is not well studied and has been found in other disorders, such as recurrent erythema multiforme. These patients have IgG antibodies to progesterone with immune complex formation and tissue deposition [7].

Given that previous research shows mast cells have extranuclear estrogen and progesterone receptors [9]**,** there is a potential for these hormones to precipitate mast cell degranulation and allergic flare symptoms, and while extranuclear progesterone receptors have been shown to induce activation of ERK and MAPK kinases [10], the role that progesterone plays in the sensitization of mast cells is ultimately unknown. 

Prior research has shown that pregnancy can trigger, exacerbate, or mitigate PH symptoms, so it is possible that high endogenous progesterone levels during pregnancy caused this patient’s sensitization [5]. Treatment of PH starts with management of the systemic allergic response, followed by exogenous progesterone at a constant dose and progesterone desensitization [11]. A study in 2017 by Heffler et al. demonstrated success in the treatment of anaphylaxis due to PH with omalizumab, a monoclonal antibody that binds to free IgE, ultimately downregulating basophils and mast cells [12]. Further therapies are determined based on the desire for conception, those undergoing fertility treatments, and those who have completed childbearing or do not wish to conceive. For those who desire conception, therapies are focused on the management of systemic allergic response with the hope that the body will desensitize to progesterone with pregnancy, followed by omalizumab [12]. For those undergoing fertility treatments, an attempt is made to desensitize the patient to coincide with the start of fertility treatments [13]. Lastly, for those who do not desire pregnancy, a trial of oral contraceptives with constant progesterone dosing has been somewhat successful and can be used unless the patient experiences anaphylaxis or skin and mucus membrane blistering [14]. If the patient does not want to bear children, therapy options include desensitization, oral contraceptives with constant progesterone dosing until menopause, gonadotropin-releasing hormone (GnRH) agonists, selective estrogen receptor modulators, and oophorectomy for those with severe PH that cannot otherwise be medically managed [14-16].

There were some challenges in this case that were unique compared to prior case reports. The lab work and ultrasound done within our gynecology office were normal, as was the workup performed by the allergist and immunologist the patient saw earlier. Unfortunately, the patient was lost to follow-up after being referred to a tertiary care center, limiting our ability to assess the efficacy of any desensitization protocol and further workup done for the patient. Additionally, serial progesterones were not performed. These may have provided better insight into the correlation of symptoms with hormonal peaks related to the menstrual cycle. As such, we were unable to find laboratory evidence confirming our clinical diagnosis of PH. Our case also demonstrated the need for further evaluation of etiologies of PH, as our patient did not report a prior exogenous progestin exposure before the onset of symptoms, which seems to be a predominant risk factor in several cases for the development of PH [16-18]. This is also an interesting case of how PH presentation can differ in patients with additional diagnoses, such as this patient’s polycystic ovary syndrome (PCOS). Patients with PCOS often have irregular menstrual cycles and hormonal irregularities, making it difficult to predict cyclical patterns [19]. This patient reported symptom onset during menses and just after, which is an unusual onset for PH. Patients with PH usually report symptoms 3-10 days prior to menstruation and approximately one to two days during menstruation [1]. The current patient’s PCOS could have caused hormonal irregularities that resulted in unusual symptom presentation, potentially making a diagnosis of PH harder to make. 

In addition, it seemed that some of the current mainstays of initial treatment of PH lost their effectiveness over time and that some treatment options, including oral contraceptive pills to regulate menstrual cycles, actually exacerbated her symptoms. While this case will add to the current literature surrounding PH, of which there are fewer than 200 documented cases [4,12], it also points to the need for further investigation into appropriate and effective treatment therapies. As the patient seemed to develop worsening systemic responses to her own progesterone and a less effective response to treatment, concerns for the patient’s mortality will remain unless optimal treatment is found and implemented. 

Our case adds to the literature regarding the theory of potential cross-sensitivity as a cause of the development of PH for those patients without prior exposure to exogenous progestin. It also provides a clinical picture of how other hormonal conditions, such as PCOS, can influence PH presentation. Our hope is that this case will add to the evidence for future exploration into the etiology of PH and in finding effective and long-term treatment of this disorder. 

## Conclusions

PH is more commonly associated with prior exogenous progestin exposure, but the patient in the current report developed symptoms in the postpartum period prior to exogenous exposure. Her allergic symptoms were progressively worsening and were exacerbated by exogenous progestin. Allergen testing was negative, but skin testing ultimately has unknown positive and negative predictive values and does not exclude a diagnosis of PH. It is also worth noting that this patient successfully carried two gestations to term without any proceeding evidence of this hypersensitivity.

Further research on the behavior of mast cells of patients with PH is necessary to better understand progesterone’s complex role in PH on systemic allergic response without prior exogenous exposure. The etiology and mechanism of progestogen sensitization are key in determining the most effective treatment options for those who do not desire sterilization or are unable to tolerate desensitization. It would also be a worthwhile endeavor to further investigate the behavior and function of mast cells in patients affected by PH to further understand their allergic manifestations. Ultimately, it is important for providers to recognize that despite its heterogeneous presentation, PH is a cyclical reaction with endogenous sensitization, and be mindful that if patients develop rashes after oral contraception initiation, they can develop exogenous sensitization.
